# Simulation of Prandtl Nanofluid in the Mixed Convective Flow of Activation Energy with Gyrotactic Microorganisms: Numerical Outlook Features of Micro-Machines

**DOI:** 10.3390/mi14030559

**Published:** 2023-02-27

**Authors:** S. S. Zafar, Ayman Alfaleh, A. Zaib, Farhan Ali, M. Faizan, Ahmed M. Abed, Samia Elattar, M. Ijaz Khan

**Affiliations:** 1 Department of Mathematical Sciences, Federal Urdu University of Arts, Sciences & Technology, Karachi 75300, Pakistan; 2 College of Engineering, Industrial Engineering Department, Umm Al-Qura University, Al-Khalidiya District, Al-Qunfudhah City 28821, Saudi Arabia; 3 Department of Industrial Engineering, College of Engineering, Prince Sattam Bin Abdulaziz University, Alkharj 16273, Saudi Arabia; 4 Industrial Engineering Department, Faculty of Engineering, Zagazig University, Zagazig 44519, Egypt; 5 Department of Industrial & Systems Engineering, College of Engineering, Princess Nourah bint Abdulrahman University, P.O. Box 84428, Riyadh 11671, Saudi Arabia; 6 Department of Mechanical Engineering, Lebanese American University, Beirut 1102 2801, Lebanon; 7 Department of Mathematics and Statistics, Riphah International University, I-14, Islamabad 44000, Pakistan

**Keywords:** nanofluid, microorganism, MHD, mixed convection, activation energy, Prandtl fluid

## Abstract

The physiological systems and biological applications that have arisen during the past 15 years depend heavily on the microscale and nanoscale fluxes. Microchannels have been utilized to develop new diagnostic assays, examine cell adhesion and molecular transport, and replicate the fluid flow microenvironment of the circulatory system. The various uses of MHD boundary flow in engineering and technology are extensive, ranging from MHD power generators and the polymer industry to MHD flow meters and pumps and the spinning of filaments. In this investigation, the (Magnetohydrodynamic) MHD flow of Prandtl nanofluid is investigated along with mixed convection, energy activation, microorganism, and chemical reaction. The flow model is considered through partial differential equations in dimensionless form which is then integrated numerically via considering the Bvp4c technique. The outcome is numerous emerging physical parameters over velocity profile, temperature, mass concentration, and microorganism with the separate pertinent quantities such as the Prandtl fluid parameter, elastic fluid parameter, magnetic field, mixed convection parameter, activation energy, chemical reaction, Brownian motion, thermophoretic force, Prandtl number, and Schmidt number. The friction factor, rate of heat transfer and Sherwood number, and density of microbes are revealed numerically and graphically. The outcomes indicate that the Prandtl fluid parameter and elastic fluid parameter tend to enhance the velocity profile. It is also noted that the Prandtl fluid parameter depreciates the thermal rate with the addition of the concentration profile while the opposite trend is recorded for activation energy. Obtained numerical outcomes are correspondingly compared with the current statistics in limiting cases and a close match is obtained.

## 1. Introduction

The term nanofluid has been of great interest to many engineers, modelers, and researchers due to its vast practical applications in solar systems, micromachine, automobiles, aerospace, electronics, and pharmaceuticals. Nanofluids are a mixture of nanoparticles and base fluids. Nanofluids are developed by suspending solid nanoparticles (having sizes between 1–100 nm) into the conventional base fluids (such as ethylene glycol, oil, and water). By adding nanoparticles to base fluids, the thermal characteristics of the fluid are enhanced. In 1995, Choi [1] experimented with the enhancement of the thermal properties by adding nanoparticles to base fluid. For convective transport, Buongiorno [2] established a mathematical model utilizing different physical constraints of nanofluid. For mass and heat transmission in a channel flow for nanoliquid, Shehzad et al. [3] presented Buongiorno’s model using (Homotopy analysis method) HAM. The mixed convective nanofluid flow past a vertical path, using a similar mathematical technique, was carried out by Xu et al. [4]. Khan and Pop [5] analyzed the flow of nanofluid over a stretchable plane and concluded that thermal conductivity is increased by Brownian movement and thermophoretic effects. Practically beneficial convective-type conditions over a vertical plane to evaluate the buoyancy-induced flow of nanoliquid were explained by Aziz and Khan [6]. In a spinning system with MHD and two-phase modeling of nanofluid, Sheikholeslami and Ganji [7] detected that convection boosts with an upsurge in Reynolds number whereas it has a reverse connection with Brownian and thermophoretic constraints. Afify and Bazid [8] explored the effects of thermophoresis diffusion and Brownian motion bounded by a vertical surface with characteristics of variable nanofluid. With the application of five different categories of nanomaterials with Marangoni convection, Lin et al. [9] observed the pseudo-plastic flow of non-Newtonian nanofluid. MHD flow of nanofluid through a stretched plane was inspected by Rashidi et al. [10]. The magnetic effect in 3D of a second-grade nanofluid past a stretched plane was probed by Hayat et al. [11]. Muhammad et al. [12,13] studied heat transfer using nanoparticles in the Ferromagnetic nanofluid. Chu et al. [14] conducted the natural convective flow of Maxwell nanofluid with influence of the Cattaneo–Christov dual theory over a stretching surface. Xiong et al. [15] reported the investigation of multiple solutions of Cross nanoliquid past a vertical thin needle. Soomro et al. [16] probed the double solution of water nanofluid using a porous cylinder. Reddy et al. [17] showed the Cross nanoliquid with a magnetic field. Ali and Zaib [18] studied Powell Eyirg nanofluid near the stagnation point. Ahmed et al. [19] conducted the Eyring Powell nanofluid due to a moving wedge. Loganathan et al. [20] carried out the Re-Eyring nanofluid. Akbar et al. [21] looked into the thermal conductivity of temperature-dependent viscosity. Akram et al. [22] scrutinized the MoS2 Rabinowitsch nanofluid inside the peristaltic flow. Ahmed et al. [23] studied the entropy production of Sutterby nanofluid. Khaled et al. [24] explained the Baffle length cavity of convection nanofluid.

Convection is a process of heat transfer in which the fluid (gas or liquid) travels away from the hot body along with the thermal energy when the temperature increases. There exist two kinds of convection, natural convection and forced convection. The combination of both types of convections is called mixed convection. It is a situation where buoyancy and pressure forces interrelate. Mixed convection has numerous applications in several industrial fields, engineering, and scientific fields, for example, chemical processes, biology, physics, geology, and many more. For fourth-grade peristaltic flow, Mustafa et al. [25] explored the solution numerically with mixed convection. Mahmood and Merkin [26] analyzed the fluid flow with mixed convection on a vertical spherical cylinder. In an absorbent surface covered with nanofluids, Ahmad and Pop [27] scrutinized the stable mixed convective flow over a smooth plane. Imtiaz et al. [28] studied the flow of mixed convective Casson fluid on the stretched cylinder using convective boundary conditions and evaluated that the percentage of mass and heat transfer increases as the thermophoretic constraint upsurges.

Non-Newtonian fluids have a vast application in industries and our daily life. A few daily life products that do not follow Newtonian law are honey, paints, toothpaste, syrup, and so on. Researchers used different models to describe the properties of non-Newtonian nanofluids; the Prandtl fluid model is one of these models. Many of the researchers discussed numerous non-Newtonian fluid models with different geometries and shapes [29,30,31,32,33]. Inside an irregular channel, Akbar [34] investigated the Prandtl fluid flow. For peristaltic mixed convective Prandtl flow of nanofluid, Hayat et al. [35] scrutinized the significance of hall current and chemical reaction. For radiation parameters, Soomro [36] analyzed the behavior of streamlines with convection at the boundary of the Prandtl flow of nanofluid through a stretching plane. With the impacts of Soret and Dufour, Hayat [37] numerically evaluated the peristalsis flow of Prandtl fluid for an endoscope. With 3D MHD flow Prandtl liquid past a horizontal surface, Kumar [38] examined the properties of mass transmission and chemical reaction. Sajid et al. [39] used the consequence of the effect of thermal radiation using Prandtl fluid flow through a variable species diffusivity with convective boundary conditions. Khan et al. [40] revealed the combined effect of stratification with heat generation of MHD Prandtl fluid.

In 1889, Svante Arrhenius (a Swedish scientist) named the minimum energy to activate a chemical reaction as Arrhenius activation energy. This energy is being used in chemical processing and engineering, lowering the temperature of nuclear reactors and recovery of thermal oil. Free convective mass transmission in a perpendicular pipe was first examined by Bestman [41] along with Arrhenius energy and chemical reaction. Through second law analysis, Zaib [42] numerically considered the MHD Casson nanomaterial flow over a wedge with Arrhenius energy and chemical process.

Nowadays, the flow of microorganisms in nanofluids attained lots of interest among scientists because of its various applications in problems regarding fluid flow. Microorganisms portray a vital role in decreasing greenhouse impact. It also has a broad variety of features in industrial and chemical processes such as preparing biofertilizers, biofuel, alcohol, and so on. Algae are rapidly growing biomass and can be transformed into biofuel or biodiesel fuel [43]. The presence of gyrotactic microorganisms in nanofluids boosts mass transfer and improves the stability of nanofluids [44] and microscale mixing. Chamkha [45] explored MHD impacts on solutal and thermal transmission across a stretched sheet. The microorganism is also effective for the optimization of the production of fibers and to evaluate the poisonousness of nanoparticles [46,47]. Khan et al. [48] studied macroscopical modeling for heat transfer of hybrid nanoliquid with MHD.

When particles in a fluid travel along a straight line, we say that the fluid is flowing laminarly. Laminar flow is a type of fluid motion in which the fluid moves in parallel layers with no lateral mixing and no disturbance between the layers. We refer to the laminar flow as a streamlined or a viscous flow. A similar idea underlies the concept of dynamic lift in aerodynamics. Wings of an airplane can rise because of a pressure difference carried by an airfoil, even when the flow around it remains laminar. Therefore, one of the uses in air mechanics is laminar flow.

A turbulent flow is a type of irregular flow that contains eddies, swirls, and flow instabilities, all of which are key concepts in the field of fluid dynamics. It is controlled by low-velocity diffusion and high-velocity convection. In contrast, the laminar regime describes the situation where fluid flows in perfectly parallel layers with no separation between them. Muhmmad et al. [49] computed the turbulence flow in FOAM.

Heat can be transmitted in three fundamental ways. Convection is a common method of heat transport in fluid mechanics, where the fluid’s motion moves the heat from one location to another. Ahmed et al. [50] conducted a numerical study of heat transfer in a rectangle. Heat transfer in the square cavities has been discussed by Mehmood et al. [51]. Some other important and valuable research on different methodologies and material analysis is listed in Refs. [52,53,54,55,56,57,58,59,60,61,62,63,64].

The core idea of this above-mentioned literature is to observe the MHD flow of mixed convective Prandtl nanofluid with activation energy in the presence of the microorganism. Activation energy has also been accounted for in the concentration equation. The flow model of ODEs is considered numerically via Bvp4c Matlab technique. The physical interpretation is graphically configured to explore the interesting features of various constraints over associated profiles. For shear force, rate of heat transfer, mass transfer, and density of microorganism, numerical results are obtained against the different values of flow constraints.

## 2. Mathematical Modeling

**Figure 1:**
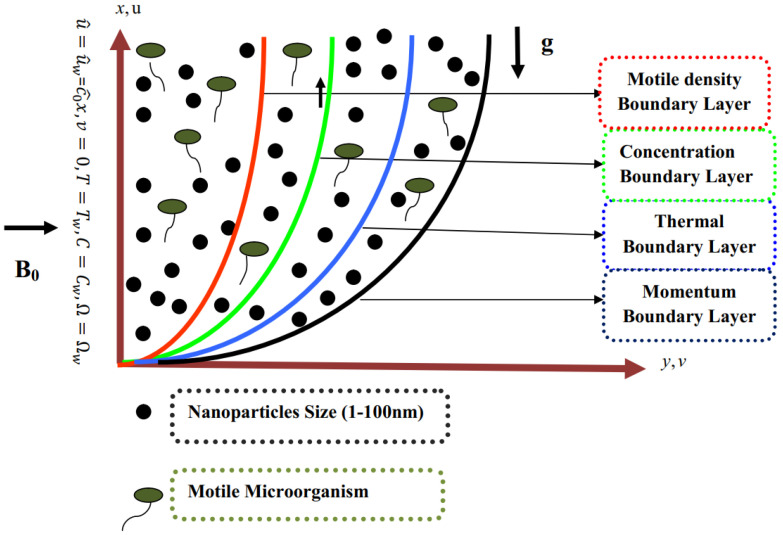
Geometry of problem.

We considered an incompressible flow of mixed convective Prandtl flow of a nanofluid in the influence of activation-energy-containing microorganisms past a stretching sheet. The velocity component was described by $\overset{⌢}{u}$ and $\overset{⌢}{v}$ in the direction of the *x* and *y*-axis, where the *x*-axis and *y*-axis were taken along the surface. Moreover, a magnetic field was implemented on the stretching sheet, as seen in Figure 1.

The extra stress tensor for the current model flow was defined as [38]

(1) $$\tau =\frac{a_{1}{Sin}^{-1}\left[\frac{1}{a_{2}}{\left\{{\left(\frac{\partial \hat{u}}{\partial y}\right)}^{2}+{\left(\frac{\partial \hat{v}}{\partial y}\right)}^{2}\right\}}^{1/2}\right]}{{\left\{{\left(\frac{\partial \hat{u}}{\partial y}\right)}^{2}+{\left(\frac{\partial v}{\partial y}\right)}^{2}\right\}}^{1/2}}\frac{\partial \hat{u}}{\partial y}$$

where $a_{1}$ and $a_{2}$ are the material variable of the Prandtl fluid model. The constitutive expressions of the MHD Prandtl nanoliquid were considered as [38]:

(2) $$\frac{\partial \hat{u}}{\partial x}+\frac{\partial \hat{v}}{\partial y}=0$$

(3) $$\left.\begin{array}{c} \hat{u}\frac{\partial \hat{u}}{\partial x}+\hat{v}\frac{\partial \hat{v}}{\partial y}=\nu \frac{a_{1}}{a_{2}}\frac{\partial^{2}\hat{u}}{{\partial y}^{2}}+\frac{\nu a_{1}}{2a_{2}^{3}}{\left(\frac{\partial \hat{u}}{\partial y}\right)}^{2}\frac{\partial^{2}\hat{u}}{\partial y}-\frac{\sigma B_{0}^{2}}{\rho }\hat{u}+g\beta \left(1-\hat{C_{\infty }}\right)\left(\hat{T}-\hat{T_{\infty }}\right)\\ -g\left[\frac{\rho_{p}-\rho_{f}}{\rho_{f}}\right]\left(\hat{C}-\hat{C_{\infty }}\right)-\gamma_{1}g\left[\frac{\rho_{m}-\rho_{f}}{\rho_{f}}\right]\left(\hat{\Lambda }-\hat{\Lambda_{\infty }}\right) \end{array}\right\}$$

(4) $$\hat{u}\frac{\partial \hat{T}}{\partial x}+\bar{v}\frac{\partial \hat{T}}{\partial y}=\alpha \frac{\partial^{2}\hat{T}}{\partial y^{2}}+\frac{\tau D_{B}}{{\hat{T}}_{\infty }}\left[{\hat{T}}_{\infty }\frac{\partial \bar{C}}{\partial y}.\frac{\partial \hat{T}}{\partial y}+\frac{D_{T}}{D_{B}}{\left(\frac{\partial \hat{T}}{\partial y}\right)}^{2}\right]$$

(5) $$u\frac{\partial \bar{C}}{\partial x}+\bar{v}\frac{\partial \bar{C}}{\partial y}=\frac{D_{T}}{{\hat{T}}_{\infty }}\left(\frac{\partial^{2}\hat{T}}{\partial y^{2}}\right)+D_{B}\frac{\partial^{2}\bar{C}}{\partial y^{2}}-K_{r}^{2}\left(\frac{\bar{T}}{\hat{T_{\infty }}}\right)Exp\left(-\frac{E_{a}}{k\hat{T}}\right)\left(\bar{C}-{\bar{C}}_{\infty }\right)$$

(6) $$u\frac{\partial \Omega }{\partial x}+\bar{v}\frac{\partial \Omega }{\partial y}+\frac{bW_{c}}{{\bar{C}}_{w}-{\bar{C}}_{\infty }}\left[\frac{\partial }{\partial y}\left(\Omega \frac{\partial \bar{C}}{\partial y}\right)\right]=D_{n}\frac{\partial^{2}\Omega }{\partial y^{2}}$$

Here, ν is the kinematic viscosity of the liquid, ρ is the electrical conductivity of the liquid, α is the thermal diffusivity of the fluid, $\tau =\frac{{\left(\rho c\right)}_{P}}{{\left(\rho C\right)}_{f}}$ is the heat capacity of the particle to the heat capacity of the fluid, $E_{a}$ is the activation energy, $D_{T}$ and $D_{B}$ is the thermophoretic and Brownian motion, and $D_{n}$ is the microorganism coefficient. The term $K_{r}^{2}{\left(\frac{T}{T_{\infty }}\right)}^{n_{1}}Exp\left(-\frac{E_{a}}{kT}\right)$ considers the Arrhenius expression that $K_{r}^{2}$ shows reaction rate, $E_{a}$ is the activation energy, $k=8.61\times 10^{-5}\mathrm{eV}K^{-1}$ and n is the fitted rate constant which generally lies in the range $-1<n_{1}<1.$

The boundary conditions were:

$$\hat{u}={\hat{u}}_{w}={\hat{c}}_{0}x,\;\bar{v}=0,T=T_{w},\;\bar{C}={\bar{C}}_{w},\;\Omega =\Omega_{w},\;at\;y=0$$

(7) $$\hat{u}\to 0,\;\hat{T}\to {\hat{T}}_{\infty },\;\bar{C}\to {\bar{C}}_{\infty },\;\Omega \to \Omega_{w}\;as\;y\to \infty$$

considering the transformations [15]:

$$\eta ={\left(\frac{\hat{c_{0}}}{\nu }\right)}^{\frac{1}{2}}y,\;\psi ={\left({\hat{c}}_{0}\nu \right)}^{\frac{1}{2}}xf\left(\eta \right),\;\hat{u}={\hat{c}}_{0}xf^{\prime }\left(\eta \right),\;\bar{\nu }={\left({\hat{c}}_{0}\nu \right)}^{\frac{1}{2}}f\left(\eta \right),$$

(8) $$\hat{T}=\left({\hat{T}}_{w}-{\hat{T}}_{\infty }\right)\theta \left(\eta \right)+{\hat{T}}_{\infty },\;\bar{C}=\left({\bar{C}}_{w}-{\bar{C}}_{\infty }\right)\phi \left(\eta \right)+{\bar{C}}_{\infty },\;\Omega =\left(\Omega_{w}-\Omega_{\infty }\right)\chi \left(\eta \right)+\Omega_{\infty }$$

With the above transformations, the continuity equation was verified and Equations (3)–(6) yielded the form:

(9) $$\alpha f^{\prime \prime \prime }-{\left(f^{\prime }\right)}^{2}+ff^{\prime \prime }+\alpha \beta {\left(f^{\prime \prime }\right)}^{2}f^{\prime \prime \prime }-Mf^{\prime }+\Lambda \left(\theta -N\phi -R_{b}\chi \right)=0$$

(10) $$\theta^{\prime \prime }+Pr\left(f\theta^{\prime }+Nb\theta^{\prime }\phi^{\prime }+Nt{\left(\theta^{\prime }\right)}^{2}\right)=0$$

(11) $$\phi^{\prime \prime }+Scf\phi^{\prime }+\frac{Nt}{Nb}\theta^{\prime \prime }-\lambda_{1}Sc{\left(1+\gamma \theta \right)}^{n}Exp\left(-\frac{E}{1+\gamma \theta }\right)\phi =0$$

(12) $$\chi^{\prime \prime }+Lbf\chi^{\prime }-Pe\left(\sigma \phi^{\prime \prime }+\chi \phi^{\prime \prime }+\chi^{\prime }\phi^{\prime }\right)=0$$

The transformed boundary conditions were [15]

(13) $$\left.\begin{array}{c} f\left(0\right)=0,\;f^{\prime }\left(0\right)=1,\;\theta \left(0\right)=1,\;\phi \left(0\right)=1,\;\chi \left(0\right)=1,\;\\ f^{\prime }\left(\infty \right)\to 0,\;\theta \left(\infty \right)\to 0,\;\phi \left(\infty \right)\to 0,\;\chi \left(\infty \right)\to 0 \end{array}\right\}$$

$$\beta =\frac{{\hat{c}}_{0}^{3}x^{2}}{2C^{2}\nu },\;\alpha =\frac{a_{1}}{a_{2}},\;Gr=\frac{g\beta \left(1-{\hat{C}}_{\infty }\right)\left({\hat{T}}_{w}-{\hat{T}}_{\infty }\right)}{c_{0}u_{w}},\;\Lambda =\frac{Gr}{{Re}_{x}^{2}},\;N=\;\frac{g\beta_{c}\left(C_{w}-C_{\infty }\right)}{g\beta_{T}\left({\hat{T}}_{w}-{\hat{T}}_{\infty }\right)},\;R_{b}=\frac{\chi_{1}\left(\rho_{m}-\rho_{f}\right)\left(\Omega_{w}-\Omega_{\infty }\right)}{\rho_{f}\beta \left(1-{\bar{C}}_{\infty }\right)\left({\hat{T}}_{w}-{\hat{T}}_{\infty }\right)},\;M=\frac{\sigma B^{2}}{{\hat{C}}_{0}\rho },\;Nb=\frac{\tau D_{B\Delta C}}{\nu },\;Pr=\;\frac{\gamma }{\alpha },\;Nt=\frac{\tau D_{t}\Delta \hat{T}}{\nu {\hat{T}}_{\infty }},\;Sc=\frac{\nu }{D_{B}},\;\lambda_{1}=\frac{K_{r}^{2}}{{\hat{C}}_{0}},\;\gamma =\frac{{\hat{T}}_{W}-{\hat{T}}_{\infty }}{{\hat{T}}_{\infty }},\;E=\frac{E_{a}}{k{\hat{T}}_{\infty }},\;\sigma =\;\frac{N_{\infty }}{N_{w}-N_{\infty }},\;Lb=\frac{\nu }{D_{n}},\;Pe=\frac{bWc}{D_{n}}.$$

Here, primes express the differentiation w.r.t η in the above terms, whereas β is the elastic parameter, α is the Prandtl fluid parameter, Gr is the Grashof number, Λ is the dimensionless mixed convective parameter, N is the buoyancy ratio parameter, Rb is the bioconvection Rayleigh number, M is the magnetic parameter, Nb is the parameter of Brownian motion, Pr is the Prandtl number, Nt is the thermophoretic parameter, Sc is the Schmidt number, $\lambda_{1}$ is the dimensionless chemical reaction constant, γ is the temperature relative parameter, E is the dimensionless energy activation, σ is the motile microbes parameter, Lb is the bioconvection Lewis number, and Pe is the Peclet number.

## 3. Quantities of Interest

The equation for surface shear stress $\tau_{w}$ and the skin friction coefficient $C_{f}$ is [58]:

(14) $$\tau_{w}=\frac{a_{1}}{a_{2}}\frac{\partial \hat{u}}{\partial y}+\frac{a_{1}}{6a_{2}^{3}}{\left(\frac{\partial \hat{u}}{\partial y}\right)}^{3},\;C_{f}=\frac{\tau_{w}}{\rho \hat{u_{w}^{2}}}$$

The expression for surface heat flux $q_{w}$ and the local Nusselt number Nu is [58]:

(15) $$q_{w}=-k\frac{\partial T}{\partial y},\;Nu_{x}=\frac{xq_{w}}{K\left(T_{w}-T_{\infty }\right)}$$

The equation for mass flux $q_{m}$ and Sherwood number $Sh_{x}$ is [58]:

(16) $$q_{m}=-D_{B}\left(\frac{\partial \bar{C}}{\partial y}\right),\;Sh_{x}=\frac{xq_{m}}{D_{n\left(C_{w}-C_{\infty }\right)}}$$

The equation for surface microorganism flux $q_{n}$ and local density number of motile microbes is:

(17) $$q_{n}=-D_{n}\left(\frac{\partial n}{\partial y}\right),\;Nn_{x}=\frac{xq_{n}}{D_{n}\left(\Omega_{w}-\Omega_{\infty }\right)}$$

Physical quantities in the dimensionless form are [58]:

μCfRex12=αf″+αβ3f‴3, NuxRex−12=−θ′(η), ShxRex−12=−ϕ′η,

(18) NnxRex−12=χ′(0)

## 4. Computational Solution

The Equations (9)–(12) including boundary conditions (13) were numerically integrated via the shooting method. First, we transformed the above system of equations into first order (initial value problem) IVP and then solved via the shooting method. Suppose

(19) $$\left.\begin{array}{c} f=\Delta_{1},\;f^{\prime }=\Delta_{2},\;f^{\prime \prime }=\Delta_{3},\;f^{\prime \prime \prime }={\Delta_{3}}^{\prime }\\ \theta =\Delta_{4},\;\theta^{\prime }=\Delta_{5},\;\theta^{\prime \prime }={\Delta_{5}}^{\prime }\\ \phi =\Delta_{6},\;\phi^{\prime }=\Delta_{7},\;\phi^{\prime \prime }={\Delta_{7}}^{\prime }\\ \chi =\Delta_{8},\;\chi^{\prime }=\Delta_{9},\;\chi^{\prime \prime }={\Delta_{9}}^{\prime } \end{array}\right\}$$

(20) $$\left.\begin{array}{c} {\Delta_{1}}^{\prime }=\Delta_{2},\\ {\Delta_{2}}^{\prime }=\Delta_{3},\\ {\Delta_{3}}^{\prime }=\frac{\Delta_{2}^{2}-\Delta_{1}\Delta_{3}+M\Delta_{2}-\Lambda \left(\Delta_{4}-N\Delta_{6}-Rb\Delta_{8}\right)}{\alpha +\alpha \beta \Delta_{3}^{2}},\\ {\Delta_{4}}^{\prime }=\Delta_{5},\\ {\Delta_{5}}^{\prime }=-Pr\left(\Delta_{1}\Delta_{5}+Nby_{5}y_{7}+Nty_{5}^{2}\right),\\ {\Delta_{6}}^{\prime }=\Delta_{7},\\ {\Delta_{7}}^{\prime }=-Sc\Delta_{1}\Delta_{7}-\frac{Nt}{Nb}\Delta_{5}^{\prime }+\lambda Sc{\left(1+\gamma \Delta_{4}\right)}^{n}Exp\left(-\frac{E}{1+\gamma \Delta_{4}}\right)\Delta_{6,}\\ {y_{8}}^{\prime }=y_{9}.\\ {\Delta_{9}}^{\prime }=-Lb\Delta_{1}\Delta_{9}+Pe\left(\sigma \Delta_{7}^{\prime }+\Delta_{7}^{\prime }\Delta_{8}+\Delta_{7}\Delta_{9}\right). \end{array}\right\}$$

Boundary conditions were transformed as

(21) $$\Delta_{1}=0,\;\Delta_{2}=\Delta_{4}=\Delta_{6}=\Delta_{8}=1.$$

Now, to solve Equations (19)–(21) using the shooting technique with boundary conditions (34), we set $\Delta_{3}=Q_{1},\;\Delta_{5}=Q_{2},\;\Delta_{7}=Q_{3}$, and $\Delta_{9}=Q_{4}$. Then, by utilizing the Bvp4c integration algorithm, the above system of ODEs was solved. The calculated solution will be convergent if absolute differences between the given and computed values of $\Delta_{3},\;\Delta_{5},\;\Delta_{7}$ and $\Delta_{9}$ are less than $10^{-5}$.

## 5. Results

**Figure 2:**
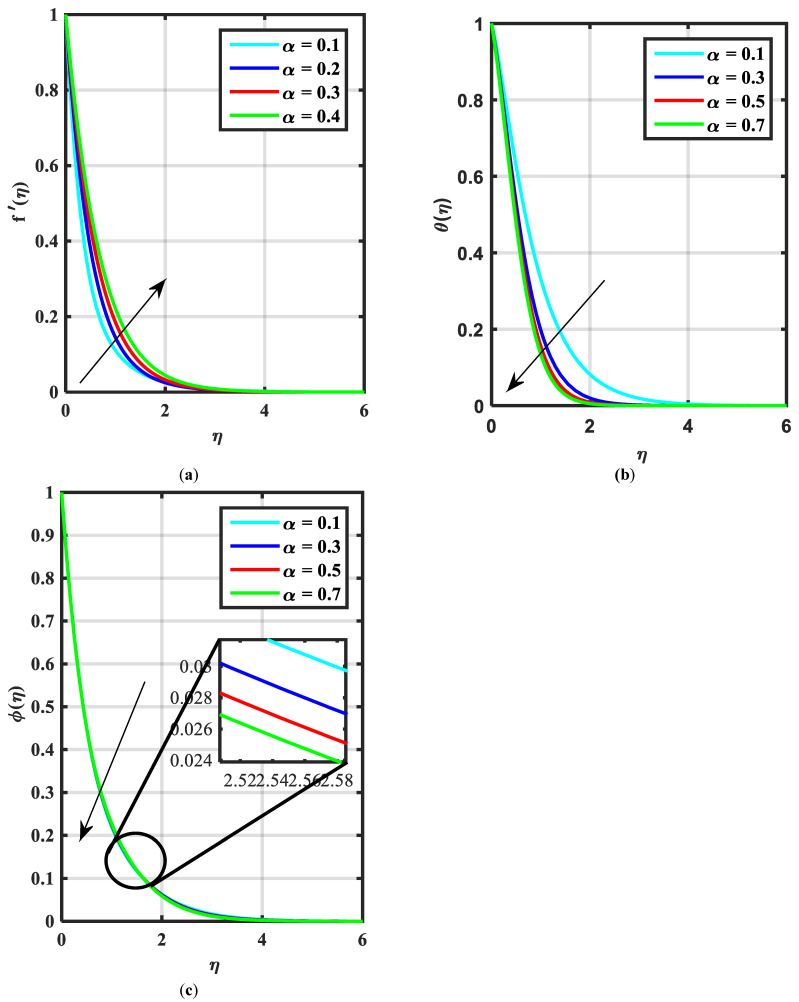
(**a**–**c**) Influence of $f^{\prime }\left(\eta \right),\;\theta \left(\eta \right)\;\phi \left(\eta \right)$ over Prandtl fluid parameter α.

**Figure 3:**
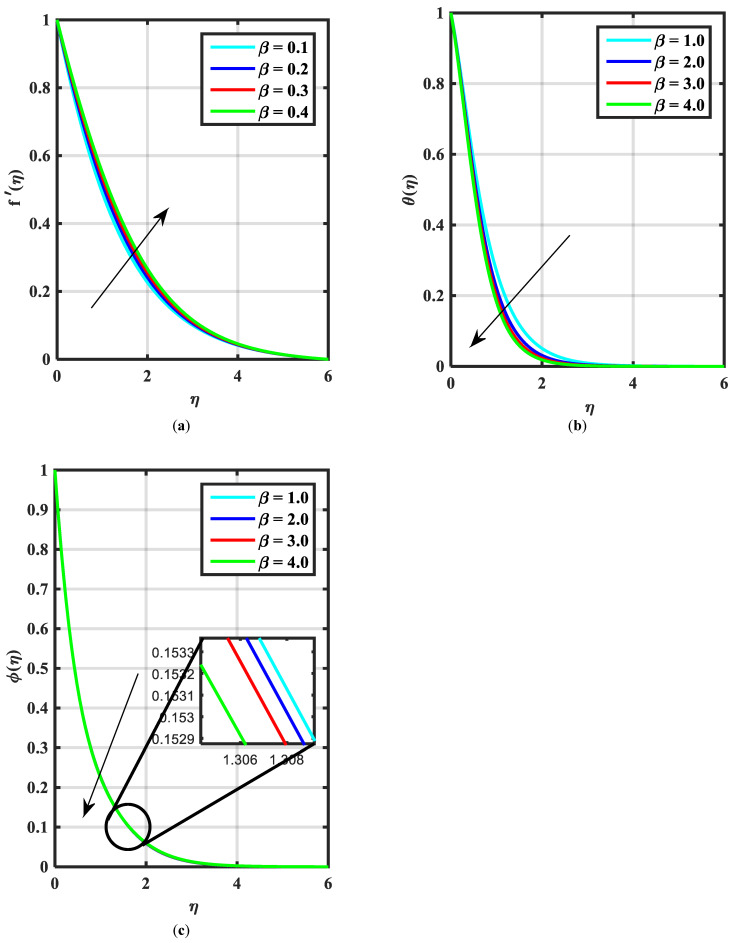
(**a**–**c**) Influence of $f^{\prime }\left(\eta \right),\;\theta \left(\eta \right)\;\phi \left(\eta \right)$ velocity over elastic parameter β.

**Figure 4:**
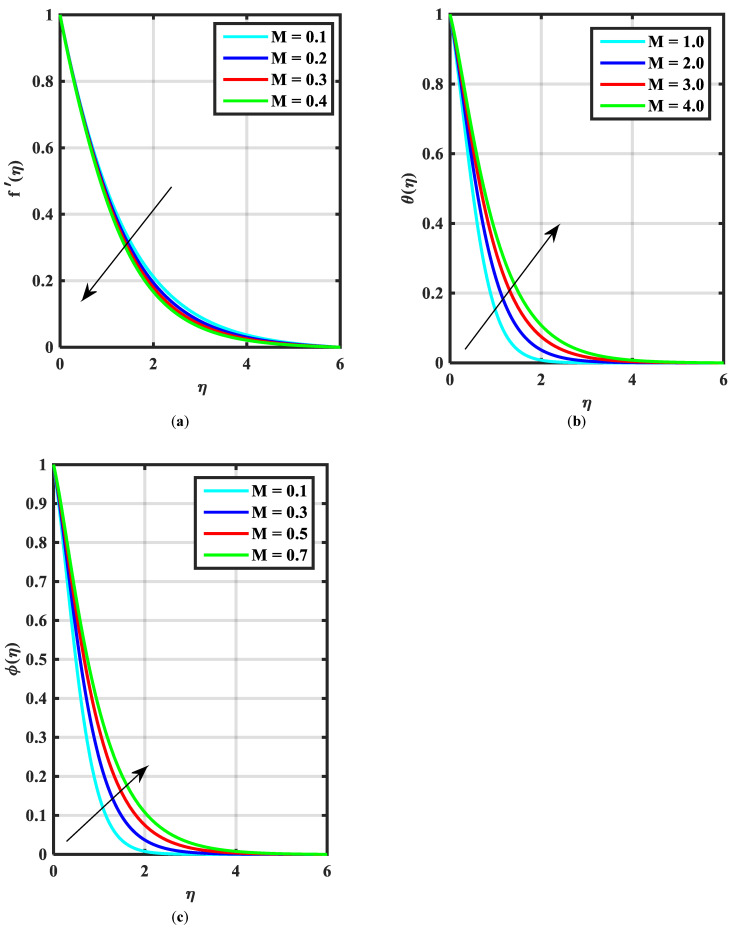
(**a–c**) Influence of $f^{\prime }\left(\eta \right),\;\theta \left(\eta \right)\;\phi \left(\eta \right)$ over magnetic parameter M.

**Figure 5:**
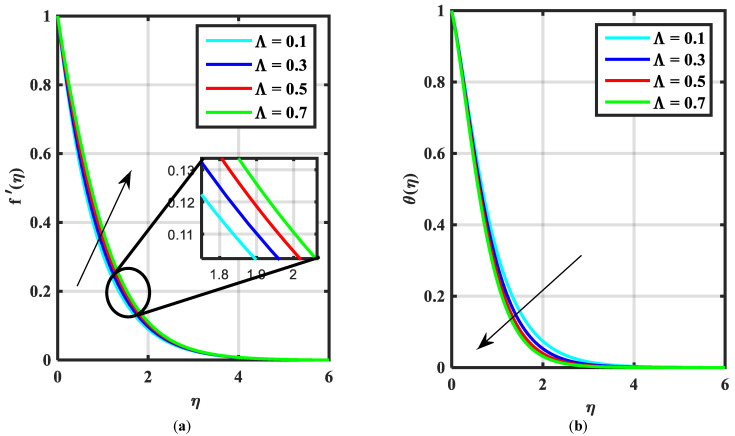
(**a**,**b**) Influence of $f^{\prime }\left(\eta \right),\;\theta \left(\eta \right)$ over mixed convective parameter ⋀.

**Figure 6:**
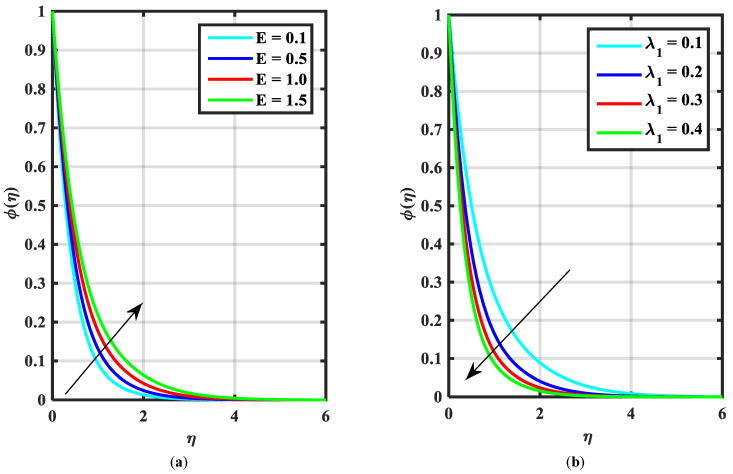
(**a**,**b**) Influence of ϕ(η) over activation energy E and chemical reaction constant $\lambda_{1}$.

**Figure 7:**
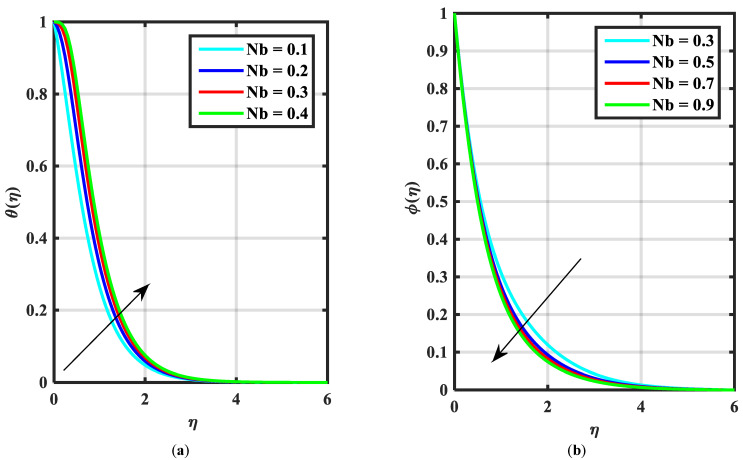
(**a**,**b**) Influence of $\theta \left(\eta \right),\;\phi \left(\eta \right)$ temperature over Brownian parameter Nb.

**Figure 8:**
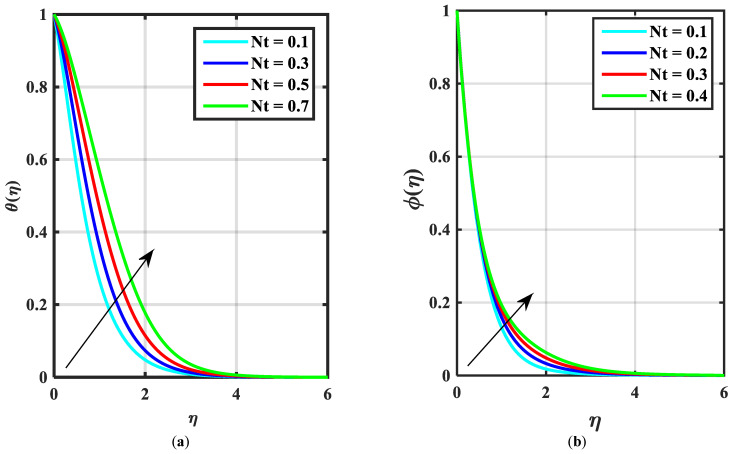
(**a**,**b**) Influence of $\theta \left(\eta \right),\;\phi \left(\eta \right)$ over thermophoretic parameter Nt.

**Figure 9:**
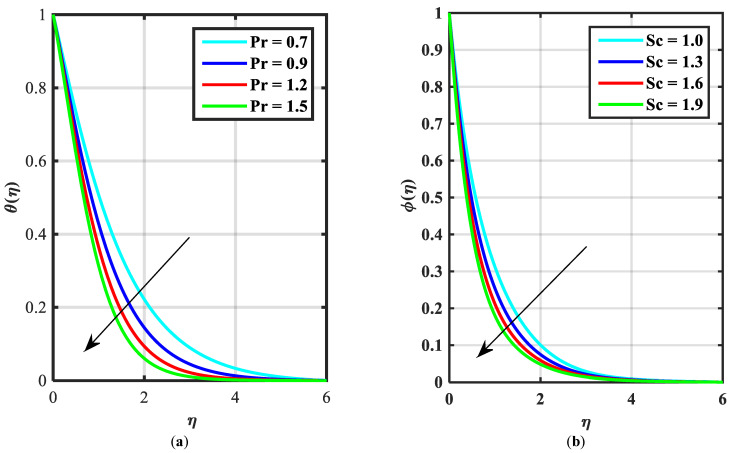
(**a**,**b**) Influence of $\theta \left(\eta \right),\;\phi \left(\eta \right)$ over Prandtl number Pr and over Schmidt number Sc.

**Figure 10:**
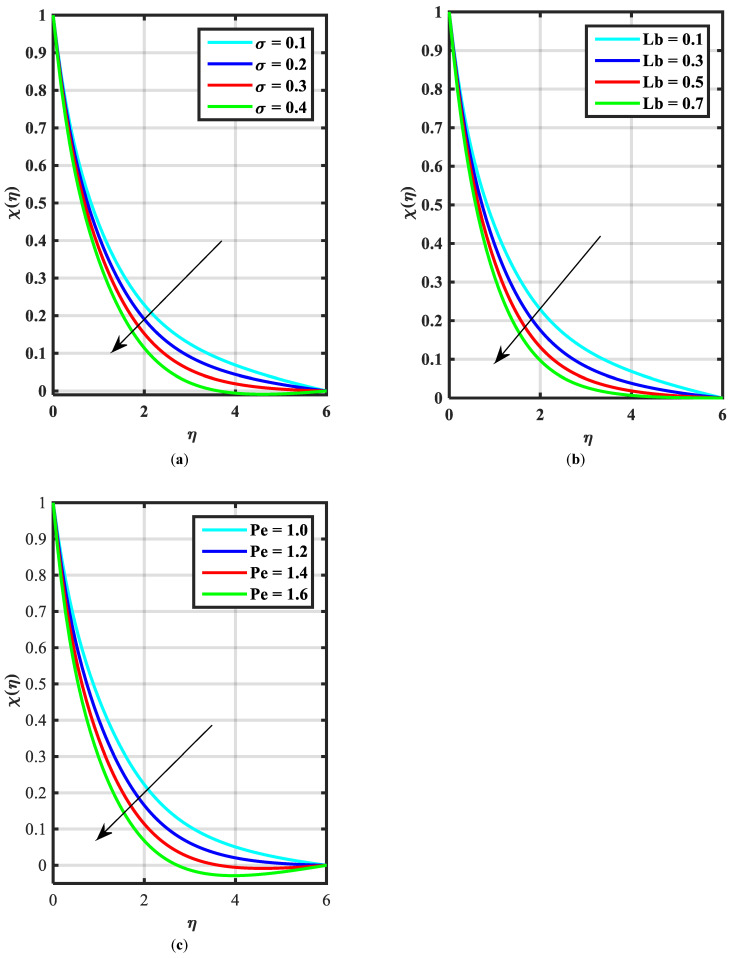
(**a**–**c**) Influence of $\chi \left(\eta \right)$ over bioconvection parameter σ, Lewis number Lb, and Peclet number Pe.

This article is devoted to expressing the impacts of Arrhenius energy over MHD microorganisms of Prandtl nanoliquid with mixed convective flow. The leading expressions were integrated numerically using the (boundary value problem) BVP4c technique. The physical behavior of various variables over $f^{\prime }\left(\eta \right),\;\theta \left(\eta \right),\;\phi \left(\eta \right),\;\chi \left(\eta \right)$ are exhibited in Figure 2, Figure 3, Figure 4, Figure 5, Figure 6, Figure 7, Figure 8, Figure 9 and Figure 10. Moreover, the numerical fluctuation in friction factor, Nusselt, Sherwood number, and microorganisms are explored through tables and graphs. Table 1 enlightens the characteristics of the drag coefficient, heat, mass, and density of microorganisms versus the parameter of fluid α against the mixed convection parameter Λ. Here, the fraction factor and mass transfer are augmented with various values of Λ and α while heat transfer rate and motile density are decayed with greater values of Λ and α. Table 2 and Table 3 reveal the validation of the $f^{\prime \prime }\left(0\right),\;\theta^{\prime }\left(0\right),\;\phi^{\prime }\left(0\right)$ in the previously published literature and found an outstanding achievement for the examination of the values of Nb, Nt, and M.

Figure 2a–c express an outcome of the Prandtl variable α over $f^{\prime }\left(\eta \right),\;\theta \left(\eta \right),$ and $\phi \left(\eta \right)$, respectively. The Prandtl fluid parameters were studied for the shear-thinning nature of the fluid. Figure 2a explains the enhancing performance of fluid parameters α on velocity $f^{\prime }\left(\eta \right)$. Physically, the fluid turns less viscous when Prandtl fluid parameter α upsurges. Thus, the velocity increases for a less-viscous fluid. Figure 2b,c reveal the impact of α on $\theta \left(\eta \right)$ and $\phi \left(\eta \right)$ profiles, respectively. The thermal layer and concentration of nanoparticles show the reducing behavior as bigger values of α. An impact of elastic parameter β over velocity $f^{\prime }\left(\eta \right)$, temperature $\theta \left(\eta \right),$ and concentration $\phi \left(\eta \right)$ are seen in Figure 3a–c, respectively. Figure 3a exhibits that the velocity field $f^{\prime }\left(\eta \right)$ enhances with the enhancing values of β. Physically, the augment of elastic dynamic viscosity results tends to resist the motion. Figure 3b,c disclose the diminishing of temperature $\theta \left(\eta \right)$ and concentration $\phi \left(\eta \right)$ for the greater values of β. Figure 4a–c view the impact of the magnetic parameter M on $f^{\prime }\left(\eta \right),\;\theta \left(\eta \right)$ and $\phi \left(\eta \right)$. Figure 4a reveals that the enhancing value of M reduces the velocity profile. Physically, a higher Lorentz force (resistive force) is produced when M increases. This resistant force converts some thermal energy into heat energy. Figure 4b,c notice that the temperature profile $\theta \left(\eta \right)$ and concentration $\phi \left(\eta \right)$ tend to increase for the greater values of M. Figure 5a,b manifest the influence of Λ on $\theta \left(\eta \right)$ and $\phi \left(\eta \right)$. From the figure, it can be seen that the improved magnitude of Λ accelerates the velocity distribution. In fact, an increase in Λ produces a larger buoyancy force due to this augmentation in the velocity of the fluid. However, the opposite trend was noted for the temperatre profile in Figure 5b. Figure 6a,b present the influence of E and chemical reaction constant $\lambda_{1}$ over $\phi \left(\eta \right)$. Figure 6a presents the increasing effect of activation energy E on the concentration of the nanoparticle. Physically, the binary chemical reaction slows down when it receives greater activation energy and a lesser temperature. Figure 6b indicates the decreasing impact of $\phi \left(\eta \right)$ by growing values of $\lambda_{1}$. Physically, the increase in the extermination process helps the liquid species dissolve effortlessly; thus, the concentration of nanoparticles decreases. Figure 7a,b amount the outcomes of Nb over the $\theta \left(\eta \right)$ and $\phi \left(\eta \right)$. For greater Nb, the temperature field escalates whereas the concentration decreases for larger Nb. Physically, the collision between the fluid particles upsurges with the growth in Nb which intensifies the temperature. Nanoparticle concentration is decayed for the greater values of Nb, as seen in Figure 7b. Figure 8a,b exhibits the growing performance of Nt temperature field $\theta \left(\eta \right)$ and concentration $\phi \left(\eta \right)$. When Nt rises, the particles of fluid transfer from hotter to cooler surfaces more rapidly which results in the rising heat and mass transfer. The influences of the Prandtl number Pr and Schmidt number Sc on θ(η) and ϕ(η) are viewed in Figure 9a,b, respectively. We detected that θ(η) and boundary layer viscosity dropped for bigger Pr. Physically, thermal diffusivity decreases with higher Pr. Therefore, the temperature of the fluid declines for larger Pr. From Figure 9b, it is clear that an escalation of Sc reduces nanoparticle concentration. This is because mass diffusivity decreases for larger values of Sc. Therefore, nanoparticle concentration declines for bigger Sc. Figure 10a–c reveal a decline in the thickness of the boundary layer of the motile density profile $\chi \left(\eta \right)$ for mounting amounts of motile microorganism parameter σ, Lewis number Lb, and Peclet number Pe.

**Figure 11:**
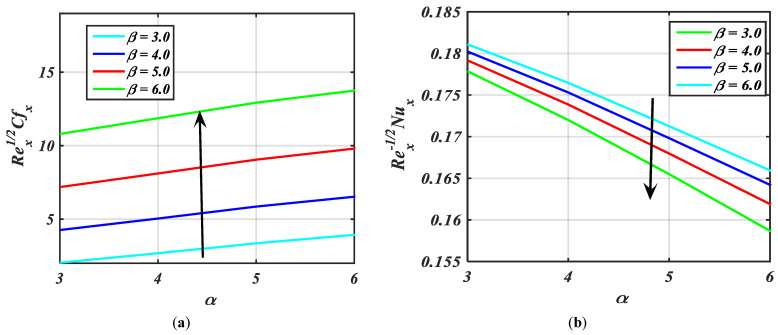

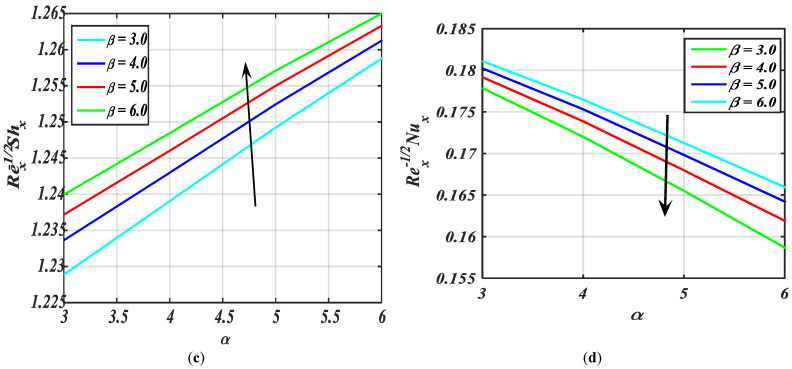
(**a**–**d**) Fluctuation in ${Re}_{x}^{1/2}{Cf}_{x},\;{Re}_{x}^{-1/2}{Nu}_{x},\;{Re}_{x}^{-1/2}{Sh}_{x},\;{Re}_{x}^{-1/2}{Nh}_{x}$ against α for diverse values of β.

The influence of β over Cf, Nu, Sh, and Nh versus fluid parameter α is portrayed via Figure 11a–d. Figure 11a,c show the increasing behavior of ${Re}_{x}^{1/2}{Cf}_{x}$ and ${Re}_{x}^{-1/2}{Sh}_{x}$ with the greater values of β. Figure 11b,d depict that ${Re}_{x}^{-1/2}{Nu}_{x}$ and ${Re}_{x}^{-1/2}{Nh}_{x}$ are the reducing function of β.

**Figure 12:**
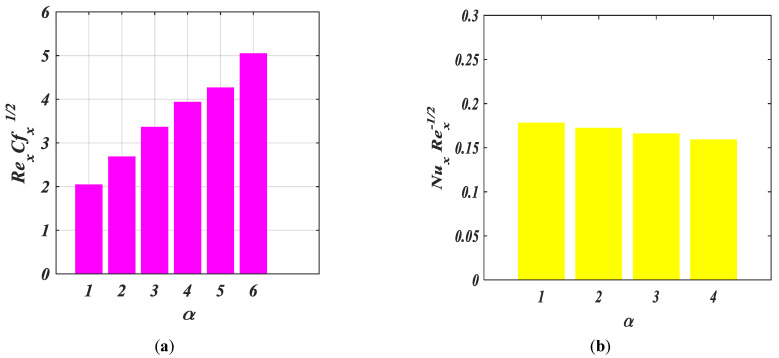

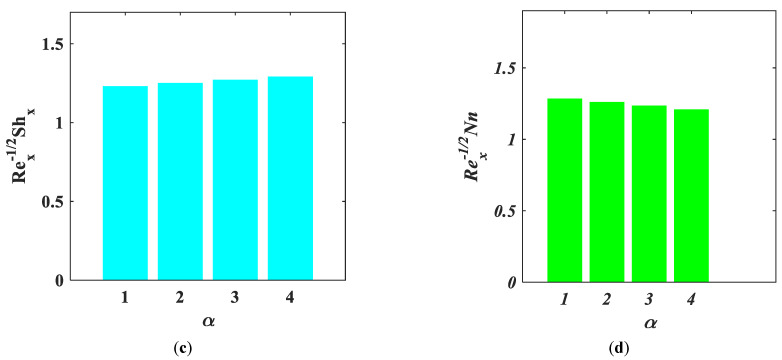
(**a**–**d**) Fluctuation in bar graphs for ${Re}_{x}^{1/2}{Cf}_{x},\;{Re}_{x}^{-1/2}{Nu}_{x},\;{Re}_{x}^{-1/2}{Sh}_{x},\;{Re}_{x}^{-1/2}{Nh}_{x}$ against α.

Figure 12a–d show the influences of drag friction, heat, mass, and motile density with various values of α.

It can be noted from these figures that ${Re}_{x}^{1/2}{Cf}_{x}$ and ${Re}_{x}^{-1/2}{Sh}_{x}$ are accelerated while ${Re}_{x}^{-1/2}{Nu}_{x}$ and ${Re}_{x}^{-1/2}{Nh}_{x}$ are decelerated with the increasing values of β.

**Figure 13:**
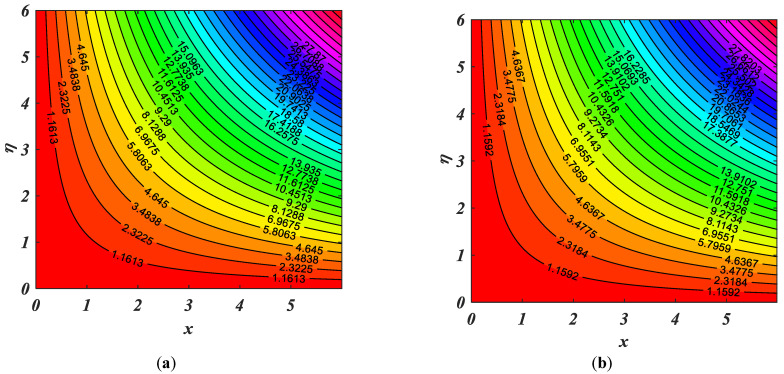
(**a**,**b**) Streamline pattern for the numerous values of (**a**) α = 1.5 and (**b**) β=2.0.

**Figure 14:**
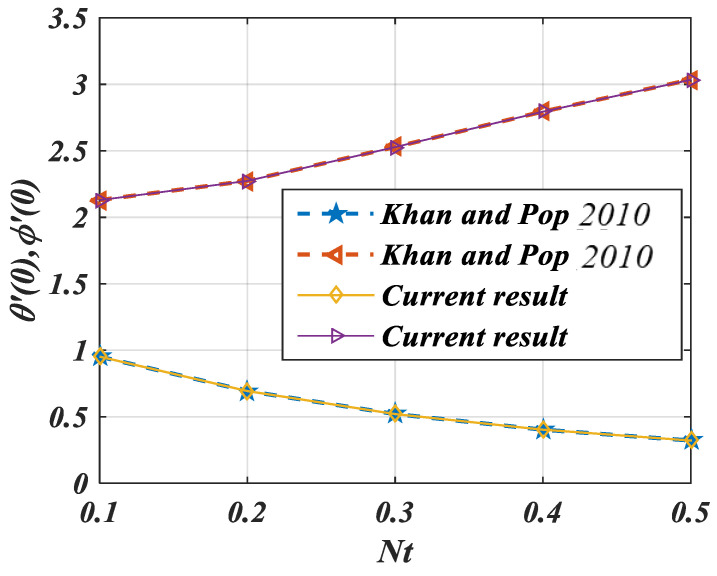
Comparison of Khan and Pop [5] and current result.

In the end, the contour patterns for α = 1.5 and β=2.0 are seen in Figure 13a,b. These figures present the stream pattern of the fluid flow at distinct values of parameters α and β. Figure 14 shows the comparisons of Khan et al. [5] and the current result.

## 6. Conclusions

In this work, we investigated the impact of nanoparticle buoyancy and activation energy on motile micro-organism flux in an MHD Prandtl fluid flow of nanofluid saturated with both small nanoparticles and gyrotactic microorganisms. The thermal conductivity, mass, and mobility microorganisms for Prandtl nanofluid flow were investigated. Nanoparticles and gyrotactic microorganisms were both part of the paradigm under consideration. Nanoparticles and buoyancy forces worked together to create bio-convective flow, which is used by microorganisms to stabilize nanoparticle suspension. We also took into account Brownian motion and thermophoretic mechanisms. The non-linear system of differential problems was obtained by invoking appropriately adapted transformations. A numerical method was employed to handle the resulting system. Below, we have outlined the most important aspects:

- Since the bio-convection slows down with higher Rayleigh numbers, the buoyancy effect of nanofluids is less, and nanoparticles cannot rise. Moreover, greater buoyancy is induced, which unquestionably opposes the fluid flow and impacts concentration. Both metrics indicate a significant decrease in flow activity.
- Prandtl fluid parameters have a noticeable impact on flow direction. Parameters of fluids have opposite effects on their viscosities. When the values of the fluid’s parameters are made greater, the fluid’s viscosity decreases, and the fluid is subsequently subject to less friction and other forms of resistance. Therefore, the fluid increases.
- For escalating values of α and β, the fluid velocity rises while the field of temperature and nanoparticle concentration declines.
- A reduction in fluid velocity occurs in the presence of nanoparticles with an increase in magnetohydrodynamic, which can be useful for medical treatments such as magnetic therapy and surgeries.
- Prandtl number Pr and Schmidt number Sc both are the reducing functions of nanoparticle concentration and temperature profile, respectively.
- For growth in the magnetic parameter, there is much stronger resistive force in the fluid motion that generates more energy dissipation and ultimately enhances the temperature of the fluid.
- For increasing amounts of Brownian diffusion Nb and thermophoretic parameter Nt, the field of temperature rises. Meanwhile, nanoparticle concentration rises because of greater Nt and diminishes for greater Nb.
- An increase in σ, Lb, and Pe (bioconvection parameters) declines density profiles and improves the diffusion rate of microbes.
- The bioconvection with nanoparticle interaction can be used in thermal transpiration for engineering and industrial processes.

## Figures and Tables

**Table 1:** Values of friction factor, Nusselt, Sherwood number, and density of microorganism versus for diverse values of Λ when M=1.0, Pr=2.0, Nt=0.1, Nb=1.5, Sc=1.0, β=6.0, γ=0.5, E=0.1, λ=1, and $n_{1}=0.2$.

α	β	Λ	***N***	$C_{f}{Re}_{x}^{1/2}$	${Nu}_{x}{Re}_{x}^{-1/2}$	${Sh}_{x}{Re}_{x}^{-1/2}$	${Nh}_{x}{Re}_{x}^{-1/2}$
3	3	1	2	2.03698	0.17786	1.22888	1.28241
		1.5		2.67861	0.17200	1.23905	1.25891
		2		3.35552	0.16550	1.24924	1.23295
		2.5		3.93163	0.15868	1.25876	1.20612
4	4	1	2.5	4.25839	0.17917	1.23363	1.28710
		1.5		5.04095	0.17386	1.24298	1.26572
		2		5.85392	0.16797	1.25244	1.24210
		2.5		6.52558	0.16190	1.26124	1.21804
5	5	1	3	7.18129	0.18023	1.23717	1.29105
		1.5		8.10691	0.17531	1.24602	1.27115
		2		9.04690	0.16982	1.25500	1.24902
		2.5		9.80026	0.16421	1.26330	1.22664
6	6	1	3.5	10.79610	0.18110	1.23994	1.29434
		1.5		11.86671	0.17647	1.24845	1.27555
		2		12.92448	0.17126	1.25709	1.25449
		2.5		13.74878	0.16595	1.26500	1.23322

**Table 2:** Comparison of numerical values for $\theta^{\prime }\left(0\right)\;and\;\phi^{\prime }\left(0\right)$ for dissimilar values of Nt when α=1 and Sc=Pr=10.

Nb = 0.1	Khan and Pop [5]		Present Result		Accuracy	
***Nt***	${-\theta }^{\prime }(0)$	${-\phi }^{\prime }(0)$	${-\theta }^{\prime }(0)$	${-\phi }^{\prime }(0)$	${-\theta }^{\prime }(0)$	${-\phi }^{\prime }(0)$
0.1	0.9524	2.1294	0.9523	2.1290	99.985%	99.9812%
0.2	0.6932	2.2740	0.6931	2.2735	99.985%	99.9780%
0.3	0.5201	2.5286	0.5200	2.5278	99.980%	99.9683%
0.4	0.4026	2.7952	0.4026	2.7941	100%	99.9606%
0.5	0.3211	3.0351	0.3210	3.0338	99.968%	99.9571%

**Table 3:** Comparison of numerical values for $f^{\prime \prime }(0)$ for dissimilar values of M when α=0 and $R_{b}=\beta =N=\Lambda =0$.

*M*	Kumar et al. [56]	Current Result	Accuracy
1.0	−1.41421	−1.41421	100%
5.0	−2.44948	−2.44949	99.9995%
10.0	−3.31662	−3.31662	100%
50.0	−7.14142	−7.14143	99.9998%
500.0	−22.3830	−22.3830	100%
1000.0	−31.6386	−31.6385	99.9996%

## Data Availability

All the data are available in the manuscript.
